# Characterization of Portable Ultra‐Low Field MRI Scanners for Multi‐Center Structural Neuroimaging

**DOI:** 10.1002/hbm.70217

**Published:** 2025-05-23

**Authors:** Emil Ljungberg, Francesco Padormo, Megan Poorman, Petter Clemensson, Niall Bourke, John C. Evans, James Gholam, Irene Vavasour, Shannon H. Kollind, Samson L. Lafayette, Carly Bennallick, Kirsten A. Donald, Layla E. Bradford, Beatrice Lena, Maclean Vokhiwa, Talat Shama, Jasmine Siew, Lydia Sekoli, Jeanne van Rensburg, Michael S. Pepper, Amna Khan, Akber Madhwani, Frank A. Banda, Mwila L. Mwila, Adam R. Cassidy, Kebaiphe Moabi, Dolly Sephi, Richard A. Boakye, Kenneth A. Ae‐Ngibise, Kwaku P. Asante, William J. Hollander, Todor Karaulanov, Steven C. R. Williams, Sean Deoni

**Affiliations:** ^1^ Department of Medical Radiation Physics Lund University Lund Sweden; ^2^ Department of Neuroimaging, Institute of Psychiatry, Psychology & Neuroscience King's College London London UK; ^3^ Hyperfine Inc Guilford Connecticut USA; ^4^ CUBRIC, Cardiff School of Psychology Cardiff University Cardiff UK; ^5^ Department of Radiology University of British Columbia Vancouver British Columbia Canada; ^6^ Department of Medicine (Neurology) University of British Columbia Vancouver British Columbia Canada; ^7^ Perinatal Imaging & Health King's College London London UK; ^8^ Division of Developmental Paediatrics, Department of Paediatrics and Child Health Red Cross War Memorial Children's Hospital Cape Town South Africa; ^9^ Neuroscience Institute University of Cape Town Cape Town South Africa; ^10^ C.J. Gorter MRI Center, Radiology Department Leids Universitair Medisch Centrum Leiden the Netherlands; ^11^ Training & Research Unit of Excellence (TRUE) Zomba Malawi; ^12^ Infectious Diseases Division International Centre for Diarrheal Disease Research Dhaka Bangladesh; ^13^ Laboratories of Cognitive Neuroscience, Division of Developmental Medicine, Department of Medicine Boston Children's Hospital Boston Massachusetts USA; ^14^ Institute for Cellular and Molecular Medicine, Department of Medical Immunology, Faculty of Health Sciences University of Pretoria Pretoria South Africa; ^15^ Department of Paediatrics & Child Health The Aga Khan University Karachi Pakistan; ^16^ University of North Carolina Global Projects Lusaka Zambia; ^17^ Botswana Harvard Health Partnership Gaborone Botswana; ^18^ Department of Psychiatry & Psychology Mayo Clinic Rochester Minnesota USA; ^19^ Department of Pediatric & Adolescent Medicine Mayo Clinic Rochester Minnesota USA; ^20^ Kintampo Health Research Centre (KHRC) Kintampo Ghana; ^21^ CaliberMRI Boulder Colorado USA; ^22^ MNCH D&T, Bill & Melinda Gates Foundation Seattle WA USA

**Keywords:** multi‐center, neuroimaging, phantom, quality control, ultra‐low field MRI

## Abstract

The lower infrastructure requirements of portable ultra‐low field MRI (ULF‐MRI) systems have enabled their use in diverse settings such as intensive care units and remote medical facilities. The UNITY Project is an international neuroimaging network harnessing this technology, deploying portable ULF‐MRI systems globally to expand access to MRI for studies into brain development. Given the wide range of environments where ULF‐MRI systems may operate, there are external factors that might influence image quality. This work aims to introduce the quality control (QC) framework used by the UNITY Project to investigate how robust the systems are and how QC metrics compare between sites and over time. We present a QC framework using a commercially available phantom, scanned with 64 mT portable MRI systems at 17 sites across 12 countries on four continents. Using automated, open‐source analysis tools, we quantify signal‐to‐noise, image contrast, and geometric distortions. Our results demonstrated that the image quality is robust to the varying operational environment, for example, electromagnetic noise interference and temperature. The Larmor frequency was significantly correlated to room temperature, as was image noise and contrast. Image distortions were less than 2.5 mm, with high robustness over time. Similar to studies at higher field, we found that changes in pulse sequence parameters from software updates had an impact on QC metrics. This study demonstrates that portable ULF‐MRI systems can be deployed in a variety of environments for multi‐center neuroimaging studies and produce robust results.

## Graphical abstract

A global study on quality control of portable, ultra‐low field MRI systems within the UNITY network. Cross‐sectional and longitudinal data are presented together with analysis of factors influencing image noise, contrast, and distortions.

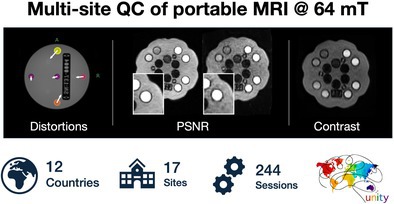

## 1 Introduction

Ultra‐low field (ULF) MRI scanners (Campbell‐Washburn et al. 2023) are currently emerging as a new category of accessible and patient‐friendly imaging systems (Sheth et al. 2021; Zhao et al. 2024; O'Reilly et al. 2021). The small size and minimal support requirements of these systems also enable portability, such as mobile scanners within a hospital for Point‐of‐Care applications (Mazurek et al. 2021), use in remote settings (DesRoche et al. 2024), or mounting the scanner in a van or small trailer (Deoni et al. 2022a). By using a low field strength (< 0.1 T) the safety profile is greatly improved with higher compatibility with medical implants due to reduced attractive forces (Van Speybroeck et al. 2021) and lower specific absorption rate (SAR) (Parsa and Webb 2023), as well as the ability to use peripheral monitoring equipment close to the scanner (Turpin et al. 2020). With a lower purchase cost and install requirements (such as no need for a Faraday cage) these systems offer new opportunities for democratizing access to MRI worldwide (Anazodo et al. 2022). At the same time, many of these advantages present novel challenges in terms of system stability. When a system operates in a non‐controlled environment or in different locations, factors such as temperature, humidity, and electromagnetic noise interference (EMI) are likely to fluctuate and could impact the image quality. This warrants studies into the stability of ULF MRI systems in real‐world settings. It should be noted that there are no standards for what constitutes low or ultra‐low field strength (Campbell‐Washburn et al. 2023). We have chosen to call 64 mT ULF here, but it should not be confused with MR systems operating at even lower field strengths utilizing, for instance, SQUID detectors which have very different operating requirements (McDermott et al. 2004).

Quality control (QC) procedures for assessing system performance are commonly used in clinical routine (American College of Radiology 2022, 2018) and in research studies (Gunter et al. 2009). Whilst QC typically involves phantom scanning, it can also be a part of in vivo data analysis pipelines to ensure acquired image data is of sufficient quality (Esteban et al. 2017). QC metrics of interest for portable ULF MRI systems overlap with those commonly studied at high field, such as signal‐to‐noise ratio (SNR), image contrast, and geometric distortions. To our knowledge, the only other study investigating the reliability of an ULF system evaluated a 50 mT portable MR system at a single site using a cylindrical phantom (Poojar et al. 2024).

In this study, we describe a framework for QC of portable ULF MRI systems, which we applied to assess initial data from scanners distributed across four continents in the UNITY Project (Ultra‐low field Neuroimaging In The Young) (Abate et al. 2024). The UNITY Project aims to study brain development in early childhood across more than 20 sites in low‐ and middle‐income countries using 64 mT MRI systems. The initial outcomes in UNITY are based on structural analysis to inform metrics such as brain volume. Since data from UNITY will be pooled from multiple sites, it is essential to investigate how image features related to structural analysis, such as SNR, contrast, and geometric distortions, vary between sites.

The objectives of this work were threefold. First, we describe the test object used for the UNITY Project, which is designed to quantify image noise, contrast, and geometric distortions. Since there are no test objects designed for 64 mT with traceable relaxation times, we begin by characterizing the T_1_ and T_2_ relaxation times in the different compartments of the phantom to determine which features correspond to tissue contrast in the brain at 64 mT. Second, we develop a framework for automated segmentation of features in the test object and analysis of quantitative QC metrics. Finally, we validate the methodology in a QC study across four continents. In this work, we will focus on QC metrics that are relevant to structural imaging, such as SNR, image contrast, and geometric distortions.

## 2 Materials and Methods

### 2.1 Phantom Design

**FIGURE 1:**
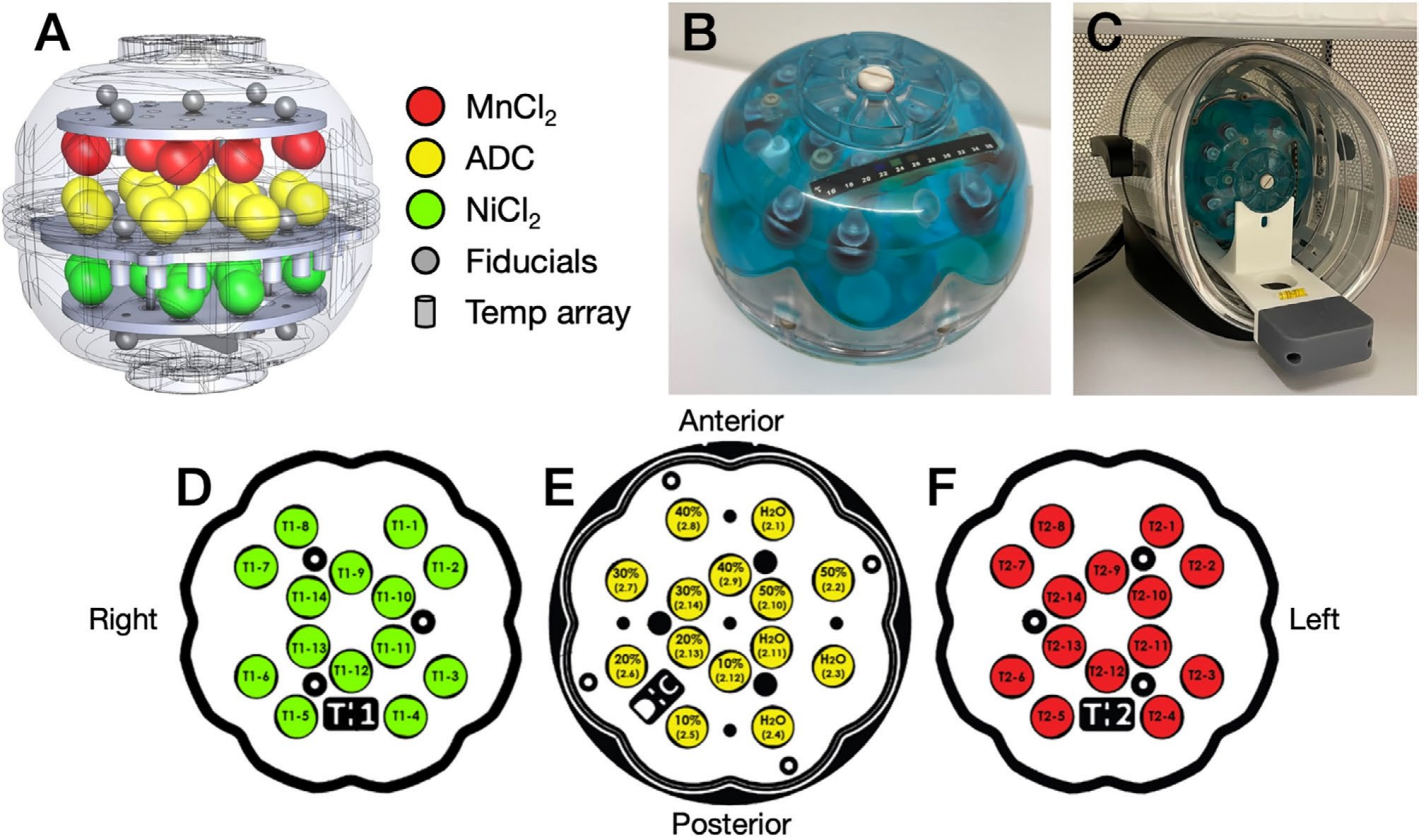
(A) The UNITY phantom configuration layout, with the MnCl_2_ spheres shown in red, the apparent diffusion constant (ADC) spheres shown in yellow, and the NiCl_2_ spheres shown in green. (B) Photograph of the phantom showing the external liquid crystal strip thermometer in black. (C) The phantom mounted in the cradle for reproducible positioning within the head coil. (D–F) Overview of the three plates containing the different tissue mimics. Each plate consists of a set of mimics, three internal supporting posts (larger black dots in triangle shape in the center of phantom), and a tag indicating the type of mimics (T_1_ for NiCl_2_ (D), DC for ADC (E), and T_2_ for MnCl_2_ (F)). The ADC plate also contains five of the geometry fiducials. Orientation labels (Left, right, anterior, posterior) indicate the orientation when mounted in the cradle.

All sites in the UNITY Project conduct their scanning on the 64 mT Swoop system (Hyperfine Inc. Guilford, CT) and have been provided with a commercially available phantom (Model 137, CaliberMRI, Boulder, CO, USA), herein referred to as the UNITY phantom. The phantom is routinely scanned following a standardized protocol, as described in Section 2.2.2. The UNITY phantom (Figure 1) is a combination of the “NIST/ISMRM” system phantom (Stupic et al. 2021) and QIBA diffusion phantom (Boss et al. 2014), but with the diameter reduced from 200 to 170 mm to approximate the head size of a 5‐year‐old child (World Health Organization 2007). The phantom consists of features to assess geometric distortion (solid fiducial spheres, resolution insert, and slice wedge), an MR‐readable thermometer (Keenan et al. 2020), and arrays for quantitative MRI features including apparent diffusion coefficient (ADC) and T_1_ and T_2_ relaxation. The quantitative MR arrays (ADC, T_1_, T_2_) consist of sets of spheres referred to as tissue mimics, where each quantitative array spans the range of healthy and diseased human tissues. Expanded descriptions of each compartment in the phantom are presented in the Supporting Information: Section 1.

The quantitative T_1_ and T_2_ arrays consist of 2 × 14 mimics with an internal diameter of 18 mm, filled with varying concentrations of NiCl_2_ (0.29–65.3 mM) and MnCl_2_ (0.0113–1.5996 mM), respectively. The purpose of these reference features is to mimic the relaxation properties of different tissues; at 3 T, the MnCl_2_ and NiCl_2_ features span a range of T_1_ and T_2_ values that covers the expected range in vivo, except for cerebrospinal fluid (MnCl_2_—T_1_: 80–2500 ms, T_2_: 5–550 ms. NiCl_2_—T_1_: 20–1900 ms, T_2_: 15–1500 ms, at 20°C). Both the NiCl_2_ and MnCl_2_ arrays cover a wide range of T_1_ and T_2_ values with the concentration ranges above, but the use of Nickel or Manganese results in different ratios between their transverse and longitudinal relaxivity (r_1_ and r_2_) (Stupic et al. 2021). The relaxivity of MnCl_2_ and NiCl_2_ changes with field strength and no reference values exist at 64 mT currently. Martin et al. performed NMR relaxometry measurements on some of the phantom mimics (Martin et al. 2023), but further measurements are needed to characterize all solutions to determine which mimics best represent white and gray matter for QC of image contrast and SNR, thus motivating the current study.

The quantitative array for ADC measurements consists of 14 spheres measuring 18 mm in internal diameter, filled with high‐purity water and polyvinylpyrrolidone (PVP) in concentrations ranging from 0% to 50% (Pierpaoli et al. 2009). The ADC values for the diffusion features range from $0.27\cdot 10^{-3}$ to $2.00\cdot 10^{-3}$ mm^2^/s (as measured at 20°C, at 3 T). Since ADC measurements in homogenous materials, such as the phantom, do not exhibit a field strength dependency, we did not perform ADC measurements. However, we characterize the T_1_ and T_2_ relaxation times in these mimics for use in future studies.

The relaxometry properties of the quantitative compartments in the phantom (T_1_, T_2_, and ADC) have a temperature dependence (Stupic et al. 2021; Martin et al. 2023), and it is therefore essential to know the temperature at the time of the scan. To accommodate for scanning environments with a wide range in temperature as expected in the UNITY project, the phantom contains both an internal liquid crystal MR‐visible (LCMRV) thermometer (Keenan et al. 2020) as well as an external liquid crystal strip thermometer with a temperature range of 16°C–36°C (Figure 1). The LCMRV thermometer consists of a set of cylinders filled with a liquid crystal, where each element contains a solution formulated for phase transition at approximate whole degree intervals between 15°C and 24°C.

**FIGURE 2:**
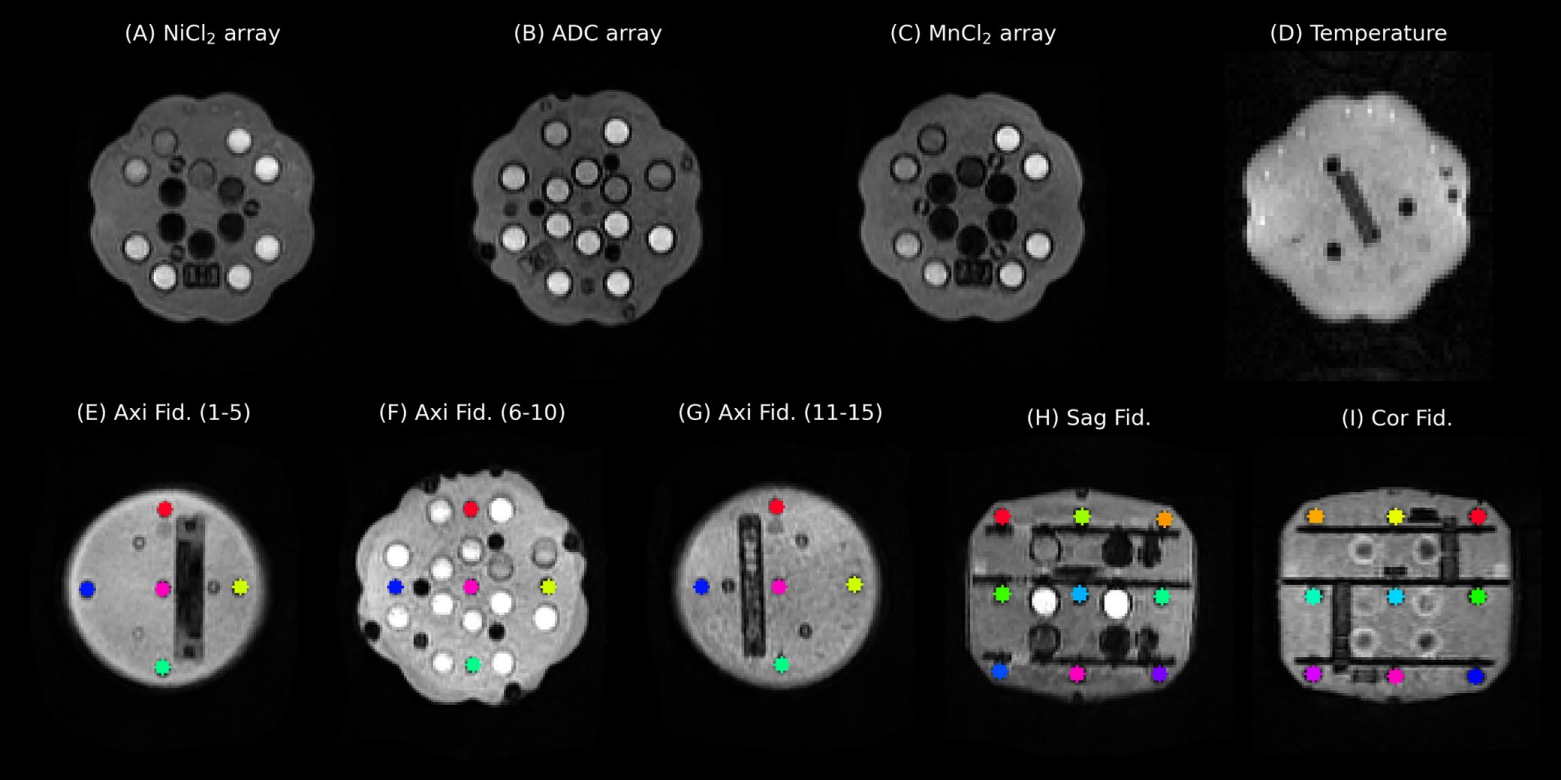
Example MR images of the UNITY phantom at 64 mT showing the different imaging features. (A–C) shows the relaxation and diffusion mimics from the axial T_2_w scan. (D) Shows the LCMRV thermometer on the gradient echo (FISP) scan where the number of dark vials, here one, indicates the temperature. (E–I) shows the 15 geometric fiducials as arranged on three axial planes. (H) and (I) shows the center fiducial planes for the sagittal and coronal scans.

The phantom also includes a set of 15 solid fiducial spheres, 10 mm in diameter, mounted in a cross pattern on each of the three plate assemblies (Figure 1). The spheres are spaced 50 mm from center‐to‐center in X, Y, and Z directions (with maximum deviation of 150 μm). The fiducial spheres provide negative contrast to the surrounding fill solution (2.9 mM CuSO_4_). The relative locations of the spheres are used to estimate geometric distortions in all three directions. Figure 2 shows example MR images of the phantom using the QC protocol proposed in this work, where the various compartments are clearly visible against the CuSO_4_ fill solution. The phantom also includes a resolution inset but this was not used in this work since the features were too small for the resolution used here.

To achieve reproducible positioning of the phantom, a cradle was developed to position the phantom within the head coil (Figure 1C). The cradle minimizes position and rotation uncertainty in all six degrees of freedom with respect to iso‐center of the scanner. However, akin to high field systems, the head coil itself is not fixed permanently inside the scanner, and residual play in its locking mechanism adds a small degree of uncertainty in the positioning of the phantom. The cradle includes a spirit level to verify that the assembly is level. To further ensure data are collected in a reproducible way at different sites, instructional videos for how to use the phantom and set up the QC protocol were recorded and shared between sites, available at www.unity‐mri.com.

### 2.2 Data Acquisition

In this work we present three sub‐studies. The first aims to characterize the T_1_ and T_2_ relaxation properties of the phantom since these have a field strength dependency. The second sub‐study aims to demonstrate the utility of the phantom in a cross‐sectional prospective study with data from 17 sites in the UNITY Project. Finally, the third sub‐study analyzes a longitudinal dataset from four of the 17 sites to investigate the stability of individual scanners over time.

All imaging experiments were carried out using 64 mT Hyperfine Inc. Swoop systems (Guilford, CT), equipped with a head coil (1‐ch TX, 8‐ch RX), and gradient set with max amplitude of 26 mT/m on Z, 25 mT/m on X and Y, and slew rate of 67 T/m/s on Z, 23 T/m/s on X and Y. A vendor‐specific integrated system for active noise correction of EMI was used on all scans. Scanner model and software level varied across the 17 sites (see summary of sites in Table 1 as well as in Supporting Information: Section 2). The sites are labeled by the phantom ID as P00XX for brevity. The materials for all the tissue mimics (MnCl_2_, NiCl_2_, and PVP) were from the same batch for all phantoms.

**TABLE 1:** Summary of sites involved in the project together with the phantom serial number that is used to identify the sites in the analysis.

Site name	Phantom	Location
CaliberMRI	P0003	Boulder, CO, United States
Lund University	P0004	Lund, Sweden
Cardiff University	P0007	Cardiff, United Kingdom
University of British Columbia	P0008	Vancouver, BC, Canada
King's College London, St Thomas Hospital	P0009	London, United Kingdom
Leiden University Medical Center	P0010	Leiden, Netherlands
Cape Universities Body Imaging Centre (CUBIC)	P0012	Cape Town, South Africa
King's College London, Centre for Neuroimaging Sciences	P0013	London, United Kingdom
Kalafong Hospital	P0014	Pretoria, South Africa
Aga Khan University	P0017	Karachi, Pakistan
Kintampo Health Research Centre	P0020	Kintampo, Ghana
Chris Hani Baragwanath Hospital	P0023	Johannesburg, South Africa
International Centre for Diarrhoeal Disease Research, Bangladesh	P0027	Dhaka, Bangladesh
Aga Khan University (field site)	P0029	Karachi, Pakistan
Women and Newborns Hospital University Teaching Hospital	P0030	Lusaka, Zambia
Botswana Harvard Health Partnership (BHP)	P0031	Gaborone, Botswana
Training & Research Unit of Excellence (TRUE)	P0039	Zomba, Malawi

#### 2.2.1 T_1_ and T_2_ Relaxation Measurements

The T_1_‐mapping experiment was carried out using a set of 20 IR‐prepared turbo spin‐echo images with a TR of 5 s and inversion times logarithmically spaced between 25 and 4000 ms. A free‐induction decay navigator with a 30° flip angle was acquired 245 ms after the turbo spin‐echo readout to track B_0_ drifts. T_1_‐weighted images were reconstructed from raw data using a non‐Cartesian conjugate‐gradient SENSE algorithm with sensitivity maps estimated using ENLIVE, all implemented in the BART toolbox (Pruessmann et al. 2001; Holme et al. 2019). The sequence used herein is the same as that used by Padormo et al. (2023), and thus the same signal equation was used for fitting the T_1_‐values, although here using phase‐corrected real‐valued data with a fitting algorithm implemented in QUIT (Wood 2017; Bydder et al. 2002). The T_2_‐mapping experiment was carried out using a multi‐echo spin‐echo sequence with 16 echoes, first TE at 9 ms, echo spacing of 5.4 ms, and TR of 3 s. Data from each echo of the T_2_‐mapping experiment were reconstructed on the scanner and T_2_‐maps were also estimated on the scanner using a single component exponential fit. To investigate the robustness and reproducibility of our results, the T_1_ and T_2_ mapping protocol was collected at two sites (P0004 and P0009), with temperature measurements using the LCMRC thermometer included at each time point. Complete details of the T_1_ and T_2_ mapping protocols are described in Table S2.

The different compartments of the phantom, that is, the tissue mimics, were automatically segmented using the method described in Section 2.3.1. Median T_1_ and T_2_ values were extracted from each region of interest (ROI) and the relaxivities (r_1_ and r_2_, where $R_{1}=\left[C\right]\cdot r_{1}+R_{1,C=0}$) were calculated using linear regression to the mimic concentration ([*C*]).

#### 2.2.2 Multi‐Site QC

Each of the 17 sites collected QC data as part of their ongoing research studies at a frequency that was manageable according to local constraints. The QC protocol was designed to assess SNR, image contrast, geometric distortions, and temperature. The protocol included five scans: T_2_w scans in all three orthogonal scan planes for estimating geometric distortions and T_2_ contrast (which is part of the UNITY in vivo protocol), a repeated T_2_w axial scan for SNR measurement, and a short TE gradient echo sequence (FISP) for assessing phantom temperature using the LCMRV thermometer. The T_2_w scans are standard sequences available on the Hyperfine system, while the short TE FISP sequence was developed for this project. Note that sites were running different software levels, resulting in differences in the default sequence parameters as well as acquisition times. The duration for the full QC protocol was approximately 10 min. A full breakdown of the sequence parameters is presented in Table S3. All sites used the custom‐built cradle for fitting the phantom inside the head coil, as described in Section 2.1. Image reconstruction for the QC data was performed directly on the scanner using the standard methods implemented by the vendor, and DICOM data was uploaded to a central repository for post‐processing.

### 2.3 Analysis of QC Metrics

#### 2.3.1 Template Based Feature Segmentation

Many of the QC metrics require labeling of different features in the phantom. The primary approach for segmentation in this work is a template‐based method which utilizes a high‐resolution “ground truth” image of the phantom obtained from high spatial resolution scans at 3 T, both using T_1_w and T_2_w sequences, with each feature manually segmented. The labels are then transferred to the acquired data using image registration, similar to atlas‐based approaches for segmentation in vivo; see Supporting Information: Section 4.1 for further details.

#### 2.3.2 Image Quality

The most commonly used proxy for image quality is SNR for which there are well‐defined measurement standards, for example, NEMA (National Electrical Manufactures Association 2014). However, multi‐coil acquisitions paired with deep learning reconstruction strategies can result in correlated noise with unknown (non‐Gaussian or Rician) distributions, which warrant alternative approaches to measure SNR (Dietrich et al. 2007). Standard methods for SNR also rely on large uniform ROIs within the phantom which are not available in the tightly packed UNITY phantom. We chose to acquire two repeated scans of the axial T_2_w images, acquired directly after each other, from which we assess image quality using Peak SNR (PSNR), a well‐established measure in the image reconstruction literature. It is typically used to compare a ground truth and reconstructed image, but here we instead employ them to compare two noisy images. Prior to computing the PSNR, both input images are normalized to have a maximum intensity of 1. The PSNR was calculated from voxels within a mask covering the phantom, obtained using the template method described above. See Supporting Information: Section 4.2 for further details and a simulated comparison between PSNR and SNR.

#### 2.3.3 Temperature

Due to the known temperature dependence of T_1_, T_2_, and ADC in the phantom, it was important to capture potential temperature changes. This is particularly relevant given the location of many of the UNITY systems, which often are in non‐temperature‐regulated hospitals, clinics, and research centers, in countries where the daytime temperature frequently exceeds 28°C. Phantom temperature was measured using the built‐in LCMRV thermometer, from images acquired by a gradient echo scan with a very short TE. Automated analysis methods were evaluated but deemed too unreliable due to variable image quality with poor definition of the LCMRV cylinders; thus, the temperature scans were read manually, and the temperature was calculated from the number of dark vials visible, Supporting Information: Section 1.3 for additional details. The maximum temperature of the LCMRV thermometer was 24.5°C; thus, any temperature higher than this will be recorded as 24.5°C.

The Larmor frequency is expected to be affected by the ambient temperature in the scan room due to the temperature dependence of the permanent magnets utilized by the 64 mT system. Assuming that the phantom and the magnet are at equilibrium temperature with the room, the phantom temperature is a good proxy for the room temperature at the start of the exam. To evaluate the relation between Larmor frequency and temperature, the center frequency was extracted from the DICOM header from each scan. The relationship between Larmor frequency and temperature was investigated using a linear mixed effects model with site as a random effect, thus allowing each site to have a different intercept to account for differences in magnet construction.

#### 2.3.4 Geometric Distortions

Geometric distortions were estimated from the position of the fiducial markers (Figure 2E–I). The fiducials were first segmented and the coordinates in the acquired image space were calculated by the center of gravity of the labels. The fiducial coordinates were then registered a common “design space” (where the fiducial positions are given by their exact reference location) using a rigid transform (Tustison et al. 2021). The distortion ($\Delta \bar{r}$) for each fiducial marker (*i*) was calculated relative to its reference location $\Delta {\bar{r}}_{i}=r_{i,\mathrm{ref}}+r_{i,\mathrm{acq}}$. Distortions larger than 20 mm were excluded as outliers due to erroneous segmentation or registration.

We chose not to use the template‐based approach to segment the fiducials, since such a method relies on co‐registration to a target and thus the position of the fiducial segmentation is constrained to a transformation field determined by the entire image. Instead, we trained a neural network with UNet design, using the nnUNet framework (Isensee et al. 2021), with training data composed of images in all slice orientations from all sites, manually segmented by a single reader. For further details regarding fiducial segmentation see Supporting Information: Section 4.3.

#### 2.3.5 Image Contrast

The NiCl_2_ and MnCl_2_ mimics were used to simulate the apparent image contrast in vivo. For each axial scan, the mimics were segmented using the template method, and the mean intensity was calculated for each mimic. Based on the relaxometry experiments, the mimics best representing adult and neonatal white and gray matter (Artz et al. 2022) were selected and the simulated white to gray matter image contrast was calculated as

$$\mathrm{Con}=\frac{S_{\mathrm{WM}}-S_{\mathrm{GM}}}{S_{\mathrm{ref}}\;}$$

where *S*_ref_ is the mean intensity of all mimics within the given array to account for global intensity scale factors.

Due to lower SNR at low magnetic field strength, image acquisitions are typically anisotropic (with 1–2 mm in‐plane resolution and 5 mm, or thicker, slices), which increases the risks of partial volume effects for the tissue contrast mimic in the slice direction. The 3D label was therefore cropped to only include the middle slice of the mimic, which was found by warping the center coordinate of the given label from the template data to the input data. Since the NiCl_2_ and MnCl_2_ mimics only are segmented to look at image contrast, they were not segmented on the sagittal and coronal scans in this work.

#### 2.3.6 Statistical Analysis

Statistical analyses were performed in Python using the statsmodels package (Seabold and Perktold 2010). We defined significant predictors to have *p* < 0.01. Several different tests were used depending on the application including linear regression, multiple linear regression, linear mixed‐effects models, and one‐way ANOVA. The PSNR variable was transformed prior to analysis as $\text{modPSNR}=10^{\text{PSNR}/10}$ to get a variable with a distribution closer to a normal distribution. For statistical models including temperature, only sessions with temperatures below the maximum of 24.5°C were included since this was the max value.

## 3 Results

### 3.1 Relaxometry Measurements

**FIGURE 3:**
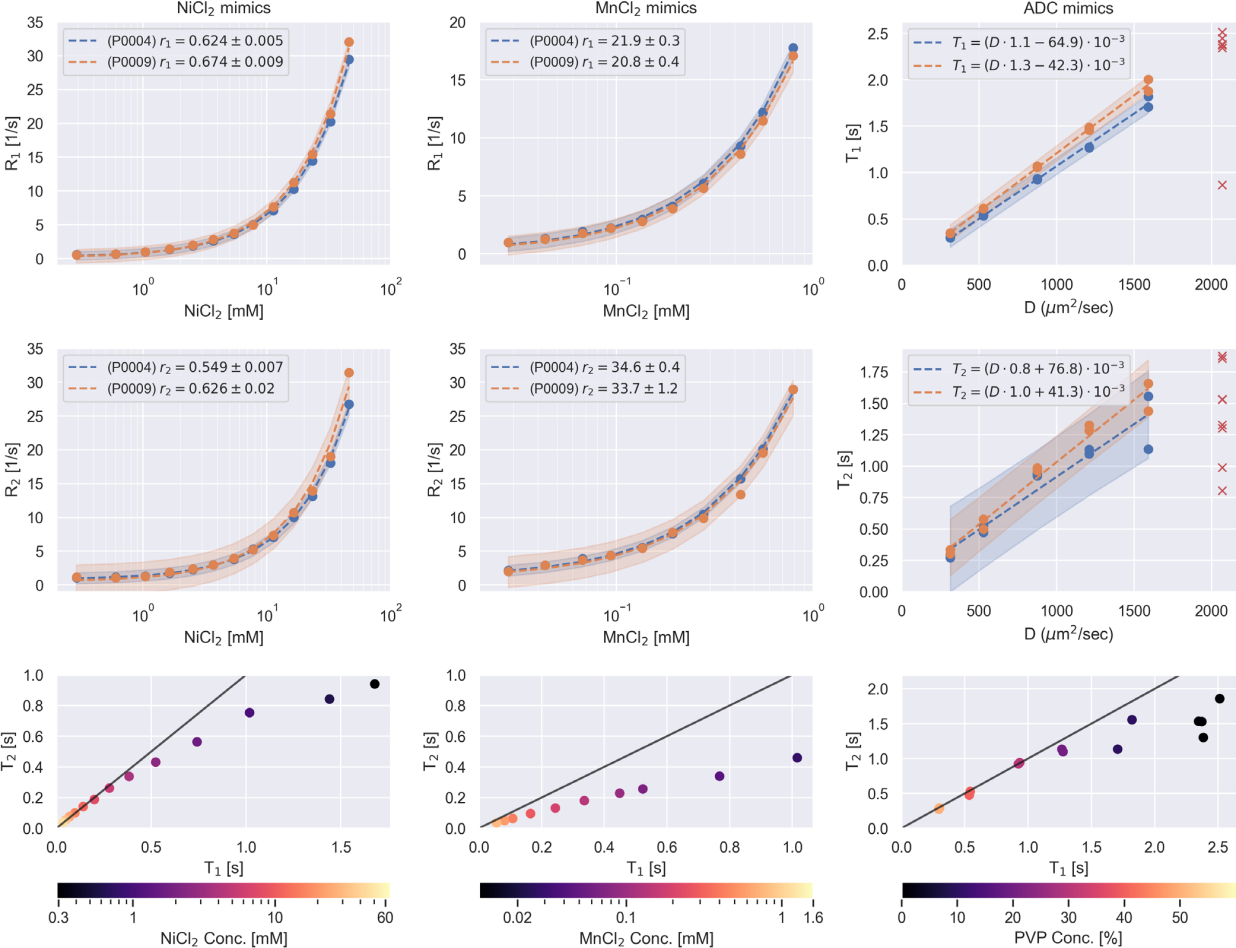
Relaxometry results for the different mimics: NiCl_2_ (left), MnCl_2_ (middle) and ADC (right). Top two rows and two left‐most columns show R_1_ and R_2_ vs. concentration of doping agent (NiCl_2_ or MnCl_2_), plotted on a logarithmic *x*‐axis to more easily visualize all the mimics. Linear regression was performed on a linear scale to obtain the relaxivity values (r_1_ and r_2_). Right column, top two rows, show T_1_ and T_2_ versus diffusivity (from NIST calibrated ADC values for each PVP mimic at 3 T, 21°C). Red crosses for the ADC mimics indicate the pure water mimics which were not included in the linear regression due to too long relaxation times for accurate measurements. Bottom row shows the T_1_/T_2_ space spanned by the mimics together with the identity line, that is, where T_1_ = T_2_, in black.

The results from the relaxometry experiments are presented in Figure 3 and Table 2 from the two sites included in the relaxometry substudy. The shortest TE and TI of the T_2_ and T_1_ mapping protocols were too long for accurate T_2_ and T_1_ measurements in the mimics with high NiCl_2_ or MnCl_2_ concentration. Therefore, we limited the relaxivity estimation to mimics with a concentration of C[NiCl_2_] < 60 mM and 0.02 < C[MnCl_2_] < 1 mM. One mimic (MnCl_2_‐2) was reported by the manufacturer to have drifted in its relaxation value and was therefore excluded (Figure S7). Comparing the relaxivity measurements (i.e., r_1_ and r_2_) between the two sites showed higher r_1_ and r_2_ for NiCl_2_, and slightly lower r_1_ and r_2_ for MnCl_2_ for site P0009. Overall, the relaxivity values measured on the MR systems in this work are similar to those found by Martin et al. at 64 mT (Martin et al. 2023), as shown in Table 2. The bottom row of Figure 3 shows that the T_2_/T_1_ ratio is close to 1 for the NiCl_2_ mimics, while it is much lower for MnCl_2_, thus better representation of brain tissue. The T_1_ and T_2_ measurements from the ADC mimics were less accurate than the NiCl_2_ and MnCl_2_ mimics, likely due to the long T_1_ and T_2_'s. Nevertheless, we found a clear linear relationship between ADC and T_1_ and T_2_, similar to previous work at high field (Pierpaoli et al. 2009). A full breakdown of the T_1_ and T_2_ values from P0004 is shown in the Tables S4–S6, together with a graphical comparison of T_1_ and T_2_ values between 64 mT and 3 T in Figure S7.

**TABLE 2:** Relaxivity values for the T_1_ and T_2_ array measured in this study compared to (Martin et al. 2023).

Scan	B_0_	T [°C]	NiCl_2_ mimics		MnCl_2_ mimics	
			r_1_ [mM^−1^ s^−1^]	r_2_ [mM^−1^ s^−1^]	r_1_ [mM^−1^ s^−1^]	r_2_ [mM^−1^ s^−1^]
P0004	64 mT	19	0.624 ± 0.005	0.549 ± 0.007	21.9 ± 0.3	34.6 ± 0.4
P0009	64 mT	23.5	0.674 ± 0.009	0.626 ± 0.02	20.8 ± 0.4	33.7 ± 1.2
Martin et al.	64 mT	20	0.59	0.57	20.46	39.28
Martin et al.	3 T	20	0.70	0.70	7.38	112.5

### 3.2 Cross‐Sectional QC Study

#### 3.2.1 Dataset Characteristics

**FIGURE 4:**
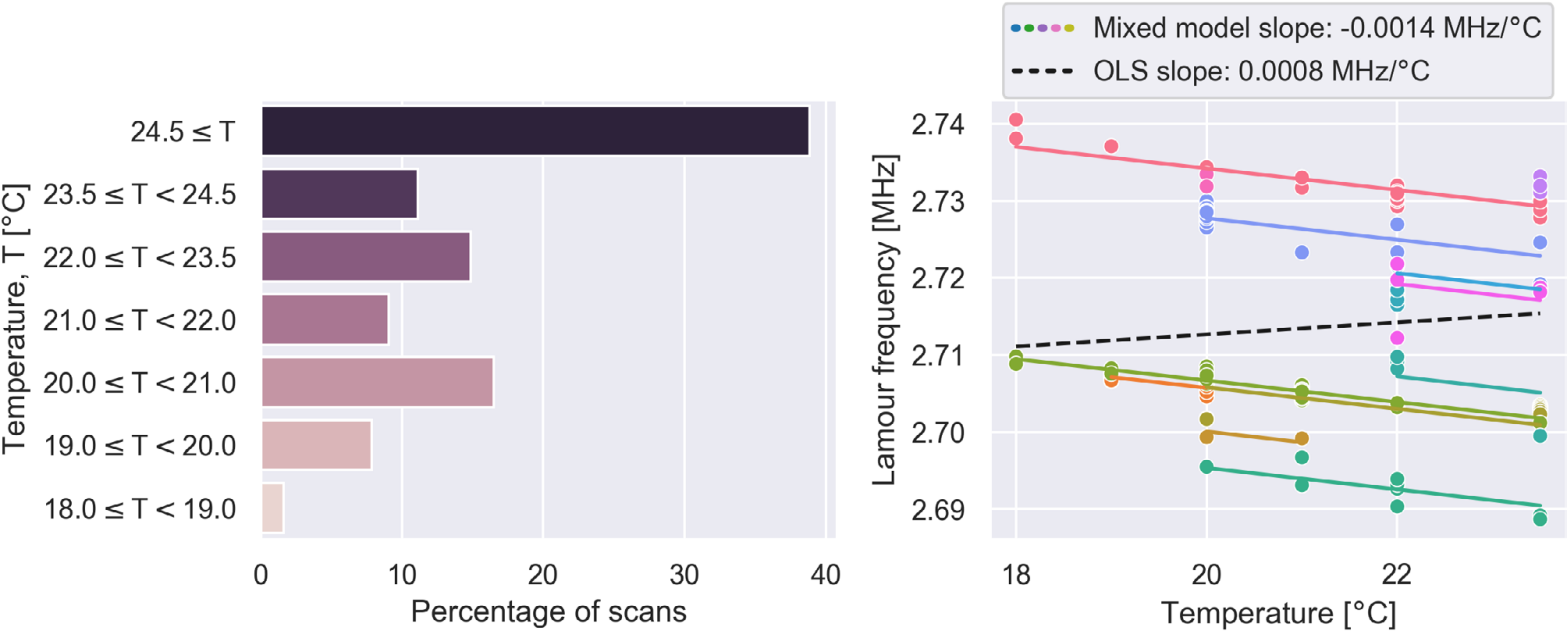
(Left) Distribution of phantom temperature at time of scan for each session. (Right) Correlation between Lamour frequency and temperature from scans with temperature readings $<24.5^{{}^{\circ}}C$. Each point represents a scan session with the color indicating the site. Data was acquired at different temperatures within sites due to normal fluctuations in the operating environment. Colored solid lines indicate linear fit obtained with the linear mixed effect model and the black dashed line is the ordinary least squares (OLS) fit to all the data.

A total of 244 scans were acquired, with one session excluded due to erroneous positioning of the phantom. See Supporting Information: Section 2 for an overview of the sites. The phantom temperature, as estimated by the internal LCMRV thermometer, ranged from 18 to ≥ 24.5°C between the sites (Figure 4). Many of the sites located in warmer climates, such as P0030 in Lusaka, Zambia, reported all scans to be ≥ 24.5°C, compared to P0004 located in Lund, Sweden, with most scans around 20°C. Almost 40% of all scans in the dataset had a recorded temperature of 24.5°C or higher.

There was a significant correlation between Larmor frequency and temperature (Figure 4) using mixed effects models with site as group variable (colored lines, slope = −0.0014 MHz/°C), but not with an ordinary least squares regression analysis to all the data (black dashed line, *p* = 0.244). This indicates that sites have a slightly different baseline Larmor frequency of the magnet, but the relative effect on the Larmor frequency by temperature changes are the same. Only scan sessions with a recorded temperature of $<24.5^{{}^{\circ}}C$ were included in the regression analysis to avoid bias from scan sessions with temperatures beyond the range of the LCMRV thermometer.

#### 3.2.2 Image Quality

**FIGURE 5:**
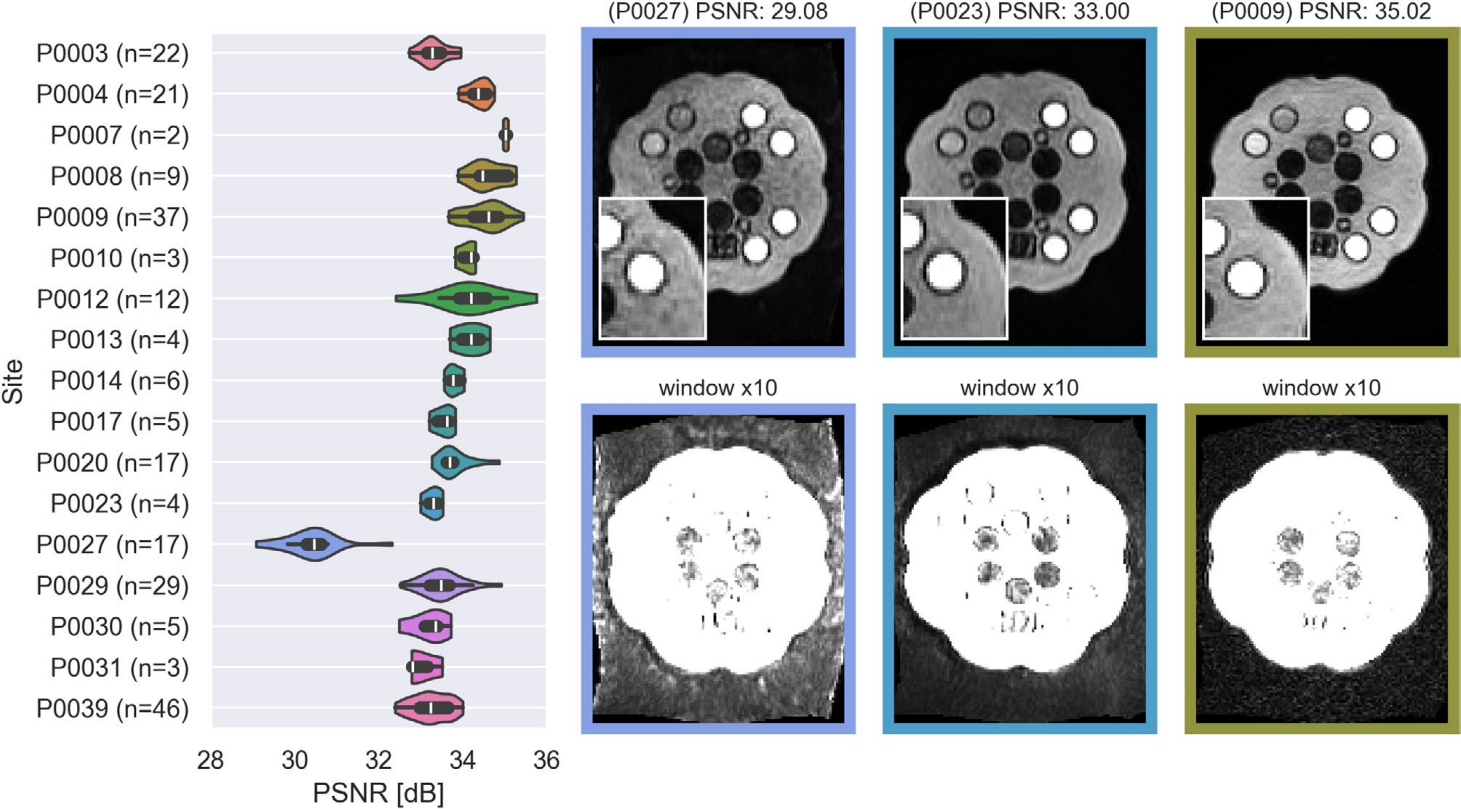
(Left) Overview of PSNR at different sites. (Right) Examples of sessions with different PSNR shown at normal window level in top row and ×10 increased brightness in bottom row to show the noise in the background. All three images shown were normalized by their mean intensity to produce a fair comparison with the same window levels.

The mean and range of PSNR were 33.6 dB [29.1, 35.8]. There were significant differences in PSNR between sites (one‐way ANOVA *p* < 1E‐10), as can also be appreciated in Figure 5. The difference in PSNR is clearly visible in the images, for instance in comparison between P0027 and P0009, shown in Figure 5. The difference in noise level is visible both in the phantom as well as in the background, shown with the increased window level in Figure 5. Multiple regression models, modeling PSNR as a function of temperature, software level (SW), and site revealed that all three explanatory variables were significant predictors.

#### 3.2.3 Image Contrast

**FIGURE 6:**
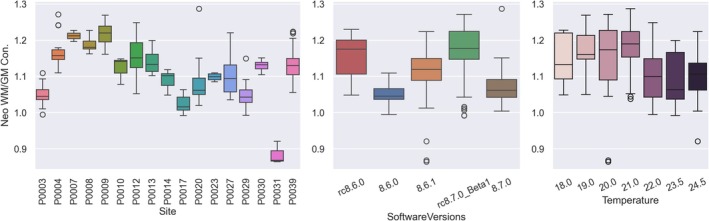
Neonatal WM/GM contrast ratio as a function of site (left), software version (middle), and temperature (right).

From the relaxation time experiment (Section 3.1), we found two MnCl_2_ mimics with T_2_ times similar to white and gray matter (WM and GM) in neonates, see Table 3. We matched the mimics only to the T_2_ times since the QC protocol uses a T_2_w sequence and thus the T_2_ relaxation time will have the strongest influence on the image contrast. The calculated WM/GM contrast varied as a function of site, SW, and temperature, as seen in Figure 6 and confirmed by a multiple regression model, which showed that contrast was significantly predicted by SW, site, temperature, and Larmor frequency.

**TABLE 3:** Mimics used to represent WM and deep GM in neonates together with the reference relaxation times (from Artz et al. 2022), the relaxation times in the mimics that were closest in T_2_, and the difference between the reference and the mimic relaxation times (ΔT_1_ and ΔT_2_).

	Mimic	Ref T_1_ [s]	Mimic T_1_ [s]	ΔT_1_ [s]	Ref T_2_ [s]	Mimic T_2_ [s]	ΔT_2_ [s]
WM	MnCl_2_‐4	0.702	0.768	0.066	0.294	0.339	0.045
Deep GM	MnCl_2_‐8	0.364	0.244	−0.121	0.139	0.131	−0.008

#### 3.2.4 Geometric Distortions

**FIGURE 7:**
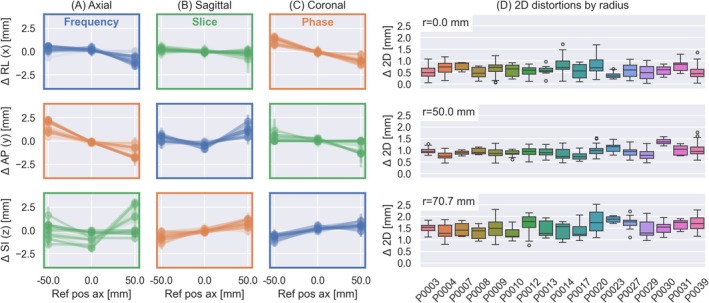
(A–C) Distortions in the RL, AP, and SI directions relative to the position of the fiducial along the same direction, shown for all three slice orientations. The encoding directions are indicated with the line color around each axis. Lines indicate individual sites. (D) Shows the 2D in‐plane (phase and frequency) distortions for all subjects organized by the distance of the fiducials from isocenter. We observe increasing distortions with distance from isocenter.

We found high reproducibility of the fiducial segmentations based on the repeated axial scans; see Figures S5–S6 with higher precision in the high‐resolution scan plane (i.e., frequency and first phase encode). Figure 7A–C shows the calculated distortion in each of the three spatial directions (RL, AP, and SI) for the three different scan planes (axial, sagittal and coronal) as a function of fiducial distance from iso‐center along the respective spatial dimension. The distortions were less than ±2.5 mm, with the exception of the slice direction, which showed larger distortions in the axial scan. We observed consistent trends with larger distortions for the distal fiducials. Figure 7D shows the magnitude of the 2D in‐plane distortions (frequency and phase encoding plane) for each site, for all three slice orientations combined, divided into three groups by the radial distance of the fiducials from iso‐center. Like in subplot Figure 7A–C, we observed that the distortions increased with greater radial distance from isocenter, with average distortions across sites of 0.594 mm at *r* = 0.0 mm (range: 0.392, 0.828), 0.926 mm at *r* = 50.0 mm (range: 0.746, 1.373), and 1.533 mm at *r* = 70.7 mm (range: 1.265, 1.873). Site, fiducial radius, and scan plane were significant predictors of the 2D distortions, but the variations between sites were very small.

**FIGURE 8:**
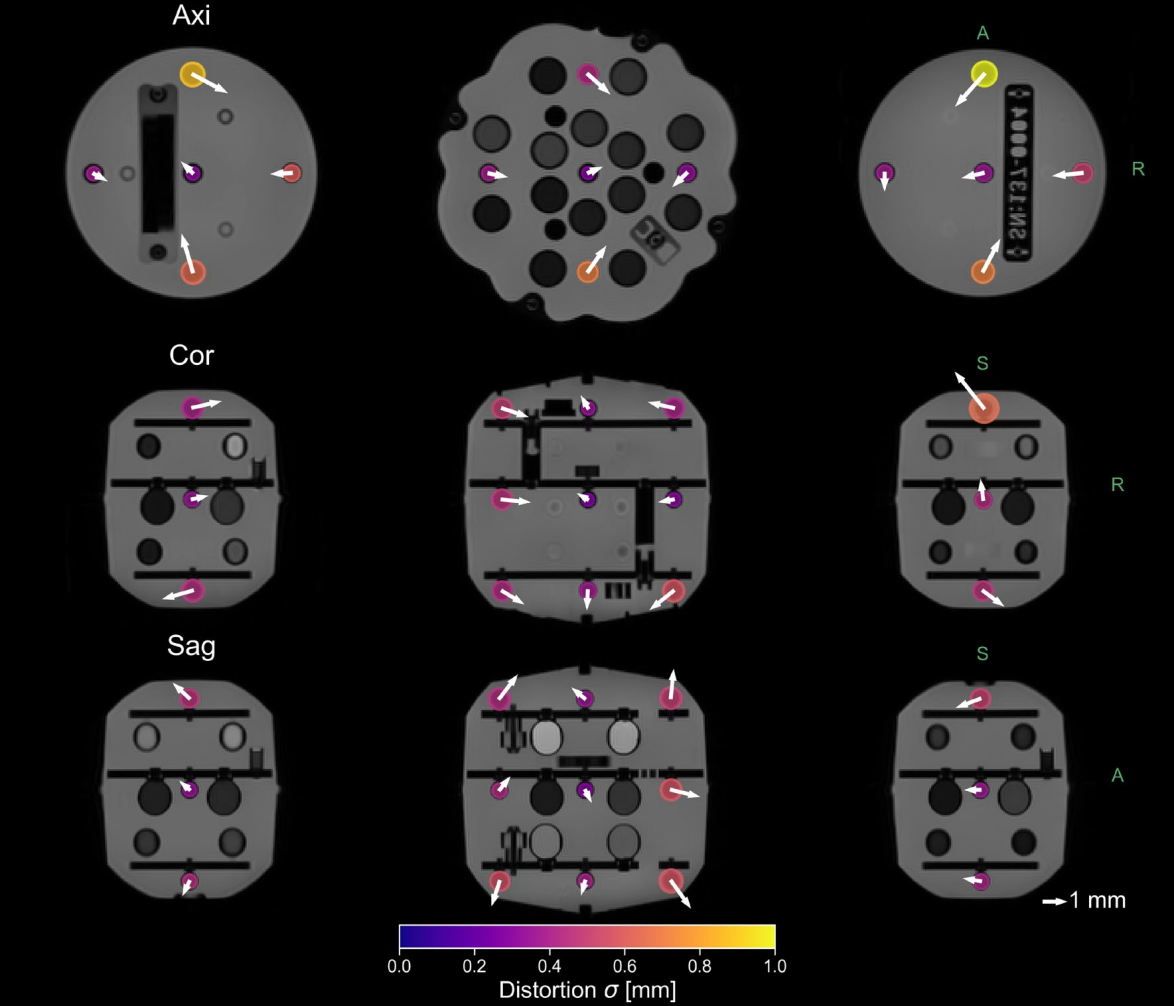
Visualization of the distortions for the three different scan orientations. The magnitude of the 2D distortion is identified by the size of the colored marker, the color of the marker indicates the standard deviation in distortion across sessions. A bright yellow marker indicates higher variability in the distortion magnitude between sites. The arrow shows the mean direction of the distortion, with the length of the arrow indicating the magnitude. Note that arrow length is scaled for visualization, reference arrow shown in figure. The image used of the phantom here is the template image used in the analysis software which was acquired at 3T.

Figure 8 shows a graphical representation of the distortions from the three scan planes with arrows indicating the magnitude and direction of the distortions for each fiducial, overlayed on a high SNR 3T scan of the phantom. Even though this visualization is averaged over all scans in the dataset, there were consistent trends in the distortions. For instance, in the axial slices, the distortions were directed inwards, while the central sagittal slice demonstrated the opposite effect.

### 3.3 Longitudinal Case Study

**FIGURE 9:**
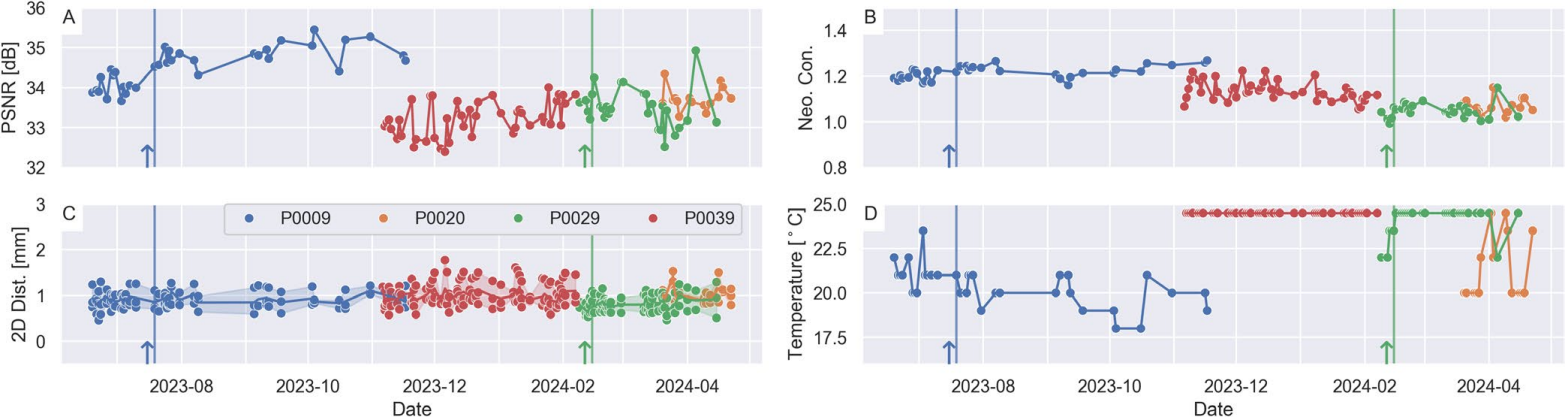
Longitudinal example from four sites showing (A) PSNR, (B) neonatal WM/GM contrast, (C) 2D distortions at 50 mm, and (D) temperature. Two of the sites had software updates performed during the longitudinal study, indicated by a vertical line and anarrow.

Longitudinal data from four sites were used to investigate longitudinal stability with data points between 2023‐11‐07 and 2024‐04‐22, Figure 9. The PSNR was highest for P0009 but the variability over time, quantified with a 95% coverage interval, was < 1 dB for all sites. The image contrast was different between sites but varied by less than 0.1 units within each site. The mean distortion, measured at a 50 mm radius from iso‐center, varied between 1.8 and 1.0 mm with variations of less than 0.5 mm for all sites. The temperature, measured with the LCMRV thermometer, was 24.5°C for all scans from P0039, while P0020 varied between 20°C and 24.5°C. Two sites (P0009 and P0029) had software updates performed during this period, which are indicated by a vertical line of the corresponding color.

## 4 Discussion

In this work we acquired and analyzed 244 phantom scans from 17 sites, representing the first project to assess image quality using a phantom on portable, ULF, MR systems on a global scale. Our results reveal a high level of stability over time and low variability between sites. A major advantage in the UNITY project is that all sites use MRI systems from the same manufacturer, thus reducing a known source of variability in multi‐center neuroimaging studies. We designed a QC protocol consisting primarily of vendor‐supplied sequences, to match the study protocol used at each specific site. As a result, we expect changes in some QC metrics between software levels where the pulse sequences have changed to improve image quality for clinical use.

The first phase of the UNITY project is focused on structural imaging outcomes with the aim of pooling data together from multiple sites. We found that the QC metrics relevant for morphometric analyses that we evaluated (PSNR, contrast, and geometric distortion) were overall stable across sites, despite large variations in operating conditions, for example, more than 6°C. There is extensive literature showing that harmonization of acquisition protocols and images is needed to reduce variability in data from multi‐center neuroimaging studies and to increase sensitivity to the target variable, such as brain growth or a specific pathology (Hu et al. 2023). While the UNITY network has reduced one confounding factor by using scanners from the same manufacturer, portable ULF MRI systems suffer from the same confounding factors as observed at high field in addition to the unique aspects of operating in variable environments.

### 4.1 Operational Environment

One difference between ULF and high‐field (> 1.5 T) systems is the physical operating environment. Portable, ULF systems have the benefit of being possible to operate in any room, but this introduces variability in temperature, humidity, and EMI. Many of the sites reported temperatures of 24.5°C, which was the maximum of the LCMRV thermometer, indicating the need to cover a wider range of temperatures in future generations of the phantom. We found temperature to be a significant predictor of both PSNR and image contrast. Changes in image contrast by temperature in the phantom could be due to the known temperature dependency of the MnCl_2_ T_2_ relaxation time (Martin et al. 2023). On the other hand, a change in PSNR with temperature is likely related to the strong correlation between scanner temperature and Larmor frequency (Figure 4). Since the electronics in the scanner are tuned for a given frequency range, changes within this range could affect the signal or noise characteristics and thus the SNR of the image.

### 4.2 Effect of Software Updates

We found that PSNR and image contrast were affected by the software version, which is to be expected. MR systems are developed for clinical applications with the goal of high diagnostic quality, and updates in software level can thus include changes in the pulse sequences. The known changes (Table S3) include timing parameters such as TE, TR, and total duration. There are also a range of sequence design choices, for example, RF pulses, gradient structures, and k‐space sampling patterns, which also affect the image, but which are not controlled by the user in normal operation mode. In fast spin echo (FSE) sequences, as used herein, changes in sequence parameters could affect the contribution of magnetization transfer (MT) and diffusion contrast. MT contrast is produced by the RF pulses and thus influenced by the RF pulse duration and amplitude, as well as the length of the spin‐echo readout (Constable et al. 1992). Diffusion contrast is produced in FSE sequences from the repeated refocusing pulses together with phase encoding and crusher gradients. However, this effect is most pronounced in sequences with refocusing flip angles $<180^{\circ }$ and at high resolutions, and thus most likely not a contributing factor in this work (Constable et al. 1992; Oakden and Stanisz 2014). Nevertheless, given the multifaceted expression of the contrast changes with software updates, standardized QC procedures offers an important and unbiased way to assess these effects to the final image.

Within the context of the UNITY project, the critical question is how changes in the image quality affect morphological measurements, for example, brain volume. Since segmentation algorithms for morphometric analysis rely on image contrast between tissues, changes in sequence parameters can result in apparent changes in the boundary between tissues and thus bias the measurement (Tardif et al. 2009; Lee et al. 2019). Image noise, on the other hand, has been shown to reduce the precision in morphological measurements but does not bias them (Tardif et al. 2009). This warrants the use of harmonization methods when combining cross‐sectional datasets from multiple sites (Wrobel et al. 2020), as well as longitudinal studies running over a long time period where software updates might be required (Takao et al. 2011).

### 4.3 Geometric Distortions

The geometric distortions were evaluated from the positions of the 15 fiducial markers, relative to their known location. Given the thick slices, we limited our analysis to the magnitude of the distortions in the high‐resolution frequency‐phase encoding plane. The distortions increased with the distance of the fiducials from the center of the phantom (Figure 7D), similar to previous work at high field (Stupic et al. 2021). While the requirements for geometric accuracy in MRI depend on the context the images are used within, the American College of Radiology (ACR) recommends a threshold of ±2 mm geometric accuracy over a 250 mm FOV (Price et al. 2015). The mean distortions at radius 70.7 mm here were 1.533 mm (range: 1.265, 1.873), indicating geometric fidelity on the same order as expected at high‐field clinical systems.

Generally speaking, distortions occur in MRI images due to non‐ideal magnetic fields. There are three different types of imperfections which can cause distortions: B_0_ field inhomogeneities, gradient non‐linearities, and eddy currents (Wang and Doddrell 2005). The degree to which images are distorted and the distortion's specific manifestation is a complex combination of all three sources and how they interact with the chosen pulse sequence (Sutton and Lam 2022). This is true both at high field and at low field. As FSE sequences are employed in this work, we expect that distortions from static field inhomogeneities are limited to the readout direction, and will be inversely proportional to the readout bandwidth (Michiels et al. 1994). These would be particularly prominent at ULF where the B_0_ field is typically more inhomogeneous (Webb and O'Reilly 2023). Non‐linearities in the image encoding gradients were historically a problem on clinical systems but are now routinely corrected for by the vendor in the reconstruction pipeline (Jack et al. 2024). We were not able to evaluate the extent to which each of these factors contribute to the distortion since we did not have access to a field mapping sequence. Nor did we have means to turn off vendor specific gradient non‐linearity correction to compare images with and without, although the vendor distortion correction is visible in Figure 5 (right panel) where the background noise profile is distorted.

While geometric distortion will affect morphological measurements, it is not trivial to map how distortions in a phantom would affect in vivo images. To fully correct an acquired image would require mapping the distortions in space across the whole field of view, as demonstrated with the ADNI phantom (Gunter et al. 2009; Maikusa et al. 2013). However, the number of fiducials in the UNITY phantom is too small for such measurement (*n* = 15 compared to *n* = 158 in the ADNI phantom). Furthermore, it is not known how novel methods combining multi‐axial images or deep learning for super‐resolution type reconstruction will affect the geometric distortions (Deoni et al. 2022b; Iglesias et al. 2022). Such approaches should preferably be applied to phantom data for QC analysis as reported here. Nevertheless, the distortions from the ULF systems in the UNITY Project were found to be small and consistent between sites and over time.

### 4.4 Quantitative Parameter Mapping

It is well established from high‐field studies (1.5 and 3T) that the T_1_ and T_2_ relaxation times change markedly in the brain during the first 1000 days of life as a result of myelination (Deoni et al. 2012). Padormo et al. 2023 studied T_1_ relaxation times in the neonatal brain at 64 mT, showing a clear reduction in T_1_ with age, in accordance with high field studies. Future iterations of the UNITY imaging protocols aim to include T_1_ and T_2_ mapping sequences to track these changes in the developing brain. The UNITY phantom would be essential in such context to ensure quantitative measurements are consistent within and between sites.

There is evidence that quantitative T_1_ and T_2_ mapping sequences are affected by pulse sequence parameters, and only by perfectly matching settings between scanners can multi‐site and multi‐scanner reproducibility be achieved (Teixeira RP et al. 2020). A quantitative reference object, such as the UNITY phantom, can ensure that the quantitative T_1_ and T_2_ values remain the same after software updates and that they are stable over time. The T_1_ and T_2_ relaxation results presented here should not be considered reference values since they are not traceable. However, our relaxometry results show promise for quantitative T_1_ and T_2_ mapping using ULF MRI as our relaxivity results were in good agreement with the NMR measurements by (Martin et al. 2023), with small differences that could be due to temperature and pulse sequence parameters.

### 4.5 Future Work

An area where we foresee improvements in future works is in instrumentation for even more granular QC analysis. For example, we were not able to obtain a pure measurement of EMI, which is known to contribute to image quality degradation in ULF MRI (Srinivas et al. 2022). Although the Swoop system has hardware and software designed to monitor and mitigate the EMI, it cannot be perfect. Residual EMI is a possible explanation of the remaining site‐to‐site variability in PSNR after accounting for temperature and software version. Future work should investigate the possibility of using an external measurement of EMI to improve our understanding of factors affecting image quality. Another example is the use of an external thermometer with a larger temperature range, as it was observed that the range of the internal thermometer in the phantom was insufficient for some of the operational environments evaluated in this study.

There are also potential improvements to the phantom. Through our relaxometry analysis we found two MnCl_2_ mimics with T_2_ values close to literature values of neonatal white and gray matter, which later were used to assess apparent image contrast. However, the T_1_ values of these mimics were not in as close correspondence to the literature, demonstrating the difficulty in designing tissue mimics with T_1_/T_2_ combinations that are similar to human tissue (Kraft et al. 2023). This is also apparent in the ADC mimics which have T_1_ and T_2_'s that are much longer than expected from human brain at 64 mT in the range of tissue‐like ADC (Adult ~1 μm^2^/ms (Choi et al. 2019), Neonate ~2 μm^2^/ms (Righini et al. 2003)). Furthermore, the resolution insert in the phantom was not used in this work since it was designed for higher resolution imaging. While the field strength does not directly influence the image resolution, the point‐spread function can be affected by system imperfections and the reduced SNR. Through the increased utility of deep learning reconstruction techniques (Koonjoo et al. 2021; Man et al. 2023), it is becoming increasingly difficult to know what the effective resolution in a given image is. This further highlights the need for standardized phantoms to evaluate the performance of imaging sequences and reconstruction methods.

## 5 Conclusions

As large multi‐center neuroimaging studies become more common in order to satisfy the need for large sample sizes, the need for QC becomes ever more pressing. We have presented a QC framework for portable ULF MR systems which builds on tools and methods previously developed for high‐field systems. With prospective data from 17 sites across four continents, we found a high degree of similarity in data quality between sites with regards to image noise, contrast, and geometric distortions. The variability we found between sites could largely be explained by differences in temperature or explicit changes in pulse sequence parameters from scanner software version updates. While the QC protocol presented herein was developed specifically for low‐field, the analysis methods can be directly translated to high‐field data. These results demonstrate that a standardized QC protocol can track changes in image quality which otherwise typically is observed in post‐processing or data harmonization. Taken together, our findings provide encouraging support for the implementation of ULF MRI for neuroimaging on a global scale.

## Conflicts of Interest

W.J.H. and T.K. are employees of CaliberMRI, the manufacturer of the phantom used in this project. F.P. and M.S.P. are employees of Hyperfine Inc. E.L. is now an employee of Philips, but this work was carried out while employed at Lund University.

## Supporting information


**Data S1.** Supporting Information.

## Data Availability

All tabular data and tools used for data analysis in this work are shared open source together with some example imaging data. **UNITY QC paper code:** https://github.com/UNITY‐Physics/unity_qa_paper — Tabular data and code for reproducing (most of) the figures in the paper. **The GHOST repository:** https://github.com/UNITY‐Physics/GHOST — The main tool used for processing the phantom data. Works together with the fiducial segmentation models and phantom 3T template linked below. **Fiducial segmentation models:** https://doi.org/10.6084/m9.figshare.26892781.v1 — Pre‐trained nnUNet models for segmenting the fiducial spheres in the UNITY phantom. **UNITY Phantom reference 3T template:** https://doi.org/10.6084/m9.figshare.26954638.v1 — High resolution template of the UNITY phantom for use with GHOST. **Example QC Data:** https://doi.org/10.6084/m9.figshare.26954056.v1 — Example QC data for UNITY acquired on the Swoop scanner at Lund University. **SOP and instructional videos:** www.unity‐mri.com/info/qa.
