# Supplementary material for: Characterization of Portable Ultra‐Low Field MRI Scanners for Multi‐Center Structural Neuroimaging
Source: Hum Brain Mapp. 2025 May 23;46(8):e70217. doi: 10.1002/hbm.70217 (PMC12099222; doi:10.1002/hbm.70217)
Supplement: Supplementary file 1 — Data S1. Supporting Information. [file HBM-46-e70217-s001.docx]

Supporting information

*Characterization of portable ultra-low field MRI scanners for multi-centre structural neuroimaging*

# Phantom details

The solutions for the tissue mimics used in the phantom have been verified at 1.5 and 3 T by the US Department of Commerce National Institute of Standards and Technology (NIST) Physical Measurement Laboratory, applying their services for “Magnetic Resonance Measurements for MRI Biomarkers” using a variable-field, variable-temperature nuclear magnetic resonance system that has been developed at NIST along with protocols and controls for verification purposes. The following sections include further details about calibration measurements done at 1.5 and 3 T on the different tissue mimics, as well as the mimic design.

## ADC – PVP array

The ADC spheres are mounted on a plate in the centre of the phantom (main document **Figure 1**) in two concentric circles with both the inner and outer ring of features containing the full range of PVP concentrations. This enables evaluation of variation in measurement of ADC as a function of distance from iso-centre. The ADC spheres have traceable calibration values from NIST, based on a pulsed gradient spin echo (PGSE) sequence over a range of b-values spanning from 0 s/mm^2^ to approximately 4956 s/mm^2^. Diffusion coefficients are derived based on a mono-exponential least squares curve fit, with uncertainty levels for the different concentration levels ranging from approximately $0.01\cdot{10}^{-3}$ to $0.05\cdot{10}^{-3}$ mm^2^/s at 21°C and 3 T.

## T1 and T2 relaxometry – NiCl_2_ and MnCl_2_ array

The NiCl_2_ and MnCl_2_ mimics have traceable calibration values from NIST at 3 T, with T_1_ measurements conducted using an inversion recovery sequence and T_2_ measurements made using a CPMG sequence. The solutions are tested over a range of temperatures which are monitored during the tests using calibrated platinum resistance thermometers (26.5°C, at 1.5 T and 16, 18, 20, 22, 24, 26°C, at 3 T). Though the NIST services are available at high field conditions only, relaxation properties of these solutions as a function of field strength have been studied both in NMR dispersion (NMRD) and MRI experiments. Bertini et al. showed that the relaxation dispersions profile of Ni_2_^+^ is constant up to ~100 MHz (~2.35 T), which was further supported by a more recent study by Martin et al. who measured relaxivity of Ni_2_^+^ and Mn_2_^+^ at 6.5 mT, 64 mT and 0.55 T [1, 2]. Mn_2_^+^ on the other hand has a more complex relaxation dispersion profile, with r_1_ of MnCl_2_ decreasing with field strength, while r_2_ increases [1].

## MR-readable thermometer

The elements of the liquid crystal MR visible (LCMRV) thermometer have a transparent visual appearance and high MR intensity when the temperature is above the transition threshold. As the temperature drops below the threshold and a phase transition occurs, the element will have an opaque visual appearance and low intensity on the MR image, see **Figure S1**. The exact relaxation properties of the LC are unknown, but we found that only a gradient echo sequence with short TE was able to visualize the different phases, suggesting short T_2_. Uncertainty in the transition points, defined based on hysteresis and other influencing factors, is typically in the range of 0.3 to 0.6 °C. The solutions for the MR-readable thermometry vials are carefully verified using Differential Scanning Calorimetry (DSC) on the phase transition points. The LCMRV vials used in this work are all from the same batch with transition temperatures listed in **Table S1**. Note that these could change on a batch-by-batch basis.


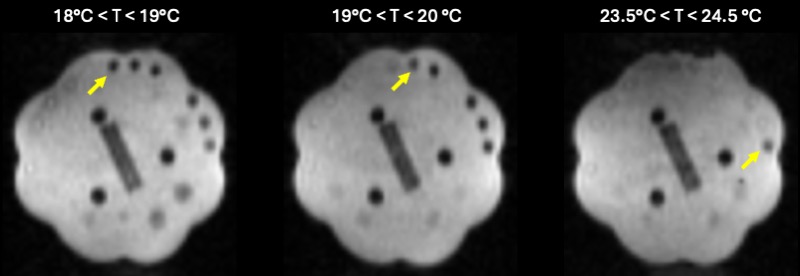


**Figure S1:** Example of thermometer readings from the internal liquid crystal (LC) thermometer. The number of dark vials indicate the temperature.

**Table S1:** Transition levels in the LCMRV thermometer for the phantom batch used in the present study.

| Vial | Transition temperature (°C) | Transition temperature uncertainty (°C) |
| --- | --- | --- |
| 1 | 14.8 | 0.3 |
| 2 | 15.8 | 0.3 |
| 3 | 16.9 | 0.3 |
| 4 | 18 | 0.3 |
| 5 | 19 | 0.4 |
| 6 | 20 | 0.5 |
| 7 | 21 | 0.5 |
| 8 | 22 | 0.5 |
| 9 | 23.5 | 0.6 |
| 10 | 24.5 | 0.6 |

## Geometric features

In addition to the fiducial spheres described in the main body of the article, the phantom also includes a slice wedge, and a resolution coupon. The resolution coupon is a hole resolution pattern, located under the ADC spheres, with reference holes ranging from 0.4 to 0.8 mm apiece, similar to the ISMRM/NIST phantom [3]. The slice wedge is a crossed wedge slice profile feature and is also mounted below the NiCl_2_ mimics, measuring 60.0 mm x 10.8 mm, with a 10° angulation. These features were not included in the current analysis since the image resolution was too low.

# Overview of sites

Figures **S2** and **S3** show an overview of when the data was collected at each site, how many scans were collected, what the distribution of temperatures were, as well as the distribution of software versions. There was a large variability in the number of scans acquired from each site for multiple reasons. Sites received their phantoms at different time points and progressed to a different extent in their adoption of a QC routine. Secondly, there are two categories of sites: those involved in methods development and those running clinical studies. It is primarily the latter group which engages in regular QC scans to quality control their in vivo data.


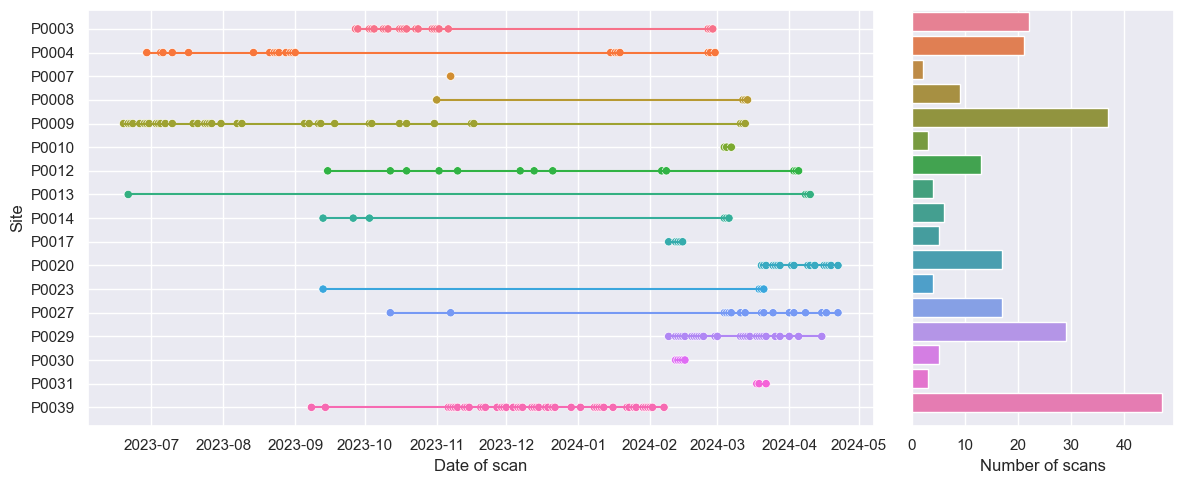


**Figure S2:** Distribution of number of scans for each site, and date of scan.


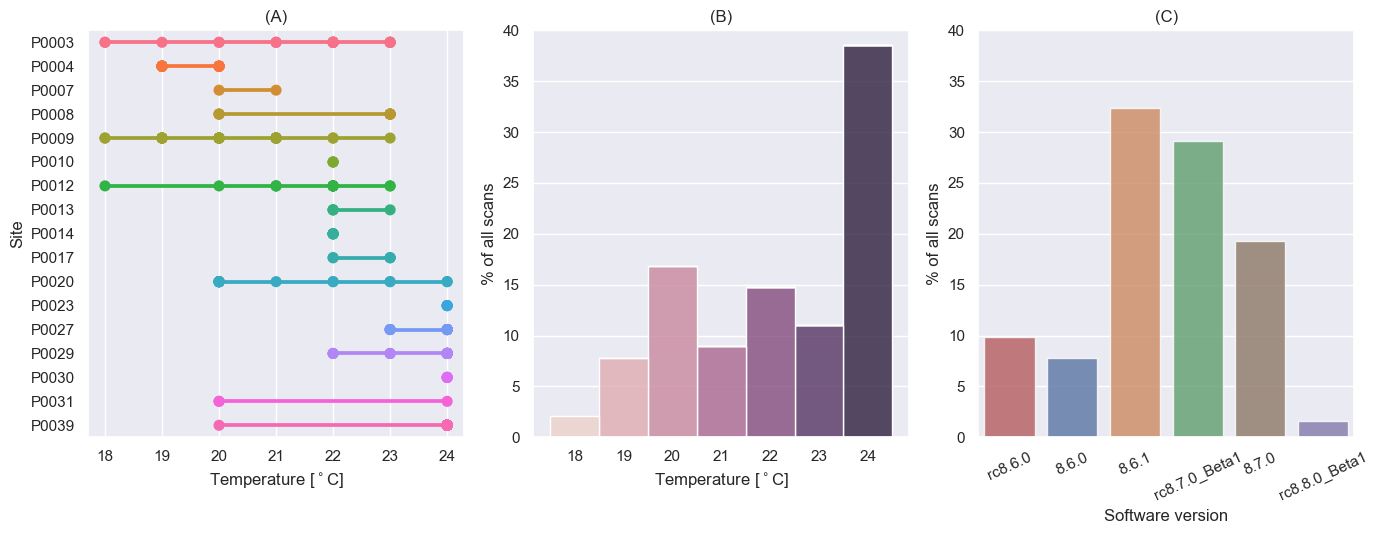


**Figure S3:** **(A)** Temperature variation between sites and **(B)** distribution of temperatures at time of scan. **(C)** Distribution of software versions.

# Sequences parameters

The sequence parameters used for the T_1_ and T_2_ mapping protocol in the relaxometry study are shown in **Table S2**. The sequence parameters used for the QC protocol are shown in **Table S3**. Not that some sequence parameters changed between software versions.

**Table S2:** Parameters for the T_1_ and T_2_ mapping protocols.

| **Parameter** | **T_1_-map** | **T_2_-map** |
| --- | --- | --- |
| **Base sequence** | FSE | FSE |
| **Resolution (mm^3^)** | 2.8x2.8x2.8 | 2x2x5 |
| **TE (ms)** | 5.04 | 18 |
| **TR (ms)** | 5000 | 5000 |
| **Duration (s)** | 20 x 530 | 5786 |
| **ETL** | 48 | 120 |
| **ESP (ms)** | 5.04 | 36 |
| **TI (ms)** | 20x log space 25-4000 | N/A |
| **TE0 (ms)** | N/A | 18 |

*Abbreviations: ESP - Echo spacing, ETL – Echo train length, TE – Echo time, TR– Repetition time, TI – Inversion time, N/A – Not applicable. *For the T_2_-mapping sequence, ESP and TE is given as the same value*

**Table S3:** Sequence parameters for QC protocol at different software levels.

| **Parameter** | **Software level** | **Temperature** | **T_2_w Axial** | **T_2_w Cor** | **T_2_w Sag** |
| --- | --- | --- | --- | --- | --- |
| **Base sequence** | **All** | **FISP** | **FSE** | **FSE** | **FSE** |
| **Resolution (mm^3^)** | **All** | 3x3x5 | 1.6x1.6x5 | 1.6x1.6x5 | 1.6x1.6x5 |
| **Matrix (PE, FE, SL)** | **All** | 60x74x36 | 112x136x40 | 112x124x36 | 136x124x36 |
| **TE (ms)** | rc8.6.0 | 0.00426 | 0.1948 | 0.2316 | 0.2384 |
|  | 8.6.0 | 0.00318 | 0.1808 | 0.22 | 0.2256 |
|  | 8.6.1 | 0.00318 | 0.1808 | 0.22 | 0.2256 |
|  | rc8.7.0_Beta1 | 0.00411 | 0.1948 | 0.2316 | 0.2384 |
|  | 8.7.0 | 0.00411 | 0.2012 | 0.2316 | 0.2384 |
|  | rc8.8.0_Beta1 | 0.00335 | 0.21723 | 0.22113 | 0.22192 |
| **TR (ms)** | rc8.6.0 | 0.00852 | 2.0 | 2.0 | 2.0 |
|  | 8.6.0 | 0.00991 | 2.0 | 2.0 | 2.0 |
|  | 8.6.1 | 0.00991 | 2.0 | 2.0 | 2.0 |
|  | rc8.7.0_Beta1 | 0.01153 | 2.0 | 2.0 | 2.0 |
|  | 8.7.0 | 0.01153 | 2.0 | 2.0 | 2.0 |
|  | rc8.8.0_Beta1 | 0.01153 | 1.6 | 1.6 | 1.6 |
| **Duration (s)** | rc8.6.0 | 45.502 | 156.619 | 138.641 | 130.663 |
|  | 8.6.0 | 52.8065 | 156.619 | 138.641 | 130.663 |
|  | 8.6.1 | 52.2705 | 156.193 | 138.193 | 130.193 |
|  | rc8.7.0_Beta1 | 61.3196 | 156.619 | 138.641 | 130.663 |
|  | 8.7.0 | 183.059 | 156.193 | 138.193 | 130.193 |
|  | rc8.8.0_Beta1 | 183.059 | 127.343 | 125.685 | 108.072 |
| **ETL** | rc8.6.0 | None | 80 | 80 | 80 |
|  | 8.6.0 | None | 80 | 80 | 80 |
|  | 8.6.1 | None | 80 | 80 | 80 |
|  | rc8.7.0_Beta1 | None | 80 | 80 | 80 |
|  | 8.7.0 | None | 80 | 80 | 80 |
|  | rc8.8.0_Beta1 | None | 78 | 78 | 76 |
| **Flip Angle** | All | 30 | 90 | 90 | 90 |
| **BW [Hz/Pixel]** | All | 800.0 | 571.429 | 516.129 | 470.588 |

*Abbreviations: ETL – Echo train length, TE – Echo time, TR– Repetition time, PE – Phase encoding, FE – Frequency encoding, SL – Slice encoding, BW – Bandwidth, FSE – Fast Spin Echo, FISP – Fast Imaging with Steady State Precession (gradient dephased non-balanced steady state free precession, also called GRASS or FFE).*

# Image analysis

## 3 T template registration

Co-registration of 64 mT phantom data to the 3T template was performed in two steps. First, an exhaustive initializer was used to find the correct orientation of the phantom using SimpleITK [4]. This was followed by a rigid and deformable registration to allow for variations in geometric distortions between the template and acquired data [5]. For implementation details, please refer to the GHOST Github repository ([github.com/UNITY-Physics/GHOST](https://github.com/UNITY-Physics/GHOST)).

## PSNR calculation

The peak signal-to-noise ratio (PSNR) is calculated as:

$$PSNR=10\log_{10} \left( \frac{R^{2}}{MSE} \right)$$

where *R* is the peak signal intensity in the images, which here is 1 since each image has been normalized to 1 before calculation, and MSE is the mean squared error between the two images. The PSNR is typically calculated between a reference and noisy image. In this work we use it to estimate the noise level in two noisy images. To validate this approach, we use the high-resolution template data described in the main body of the paper and generated noisy image data with the same resolution as the ULF image data, by adding Rician noise to pairs of images. We then compared this to calculating the PSNR between a noise free and noisy image as well (the traditional use of the metric). **Figure S4** shows that calculating PSNR between two noisy images produces very similar results to calculating it between a noise free reference and noisy image. This figure also shows a comparison to the standard SNR measurement with the dual acquisition method, which has a different offset and shape but still follows the added noise level well. The code to reproduce these results can be found in the github repository linked in the main body of the paper.


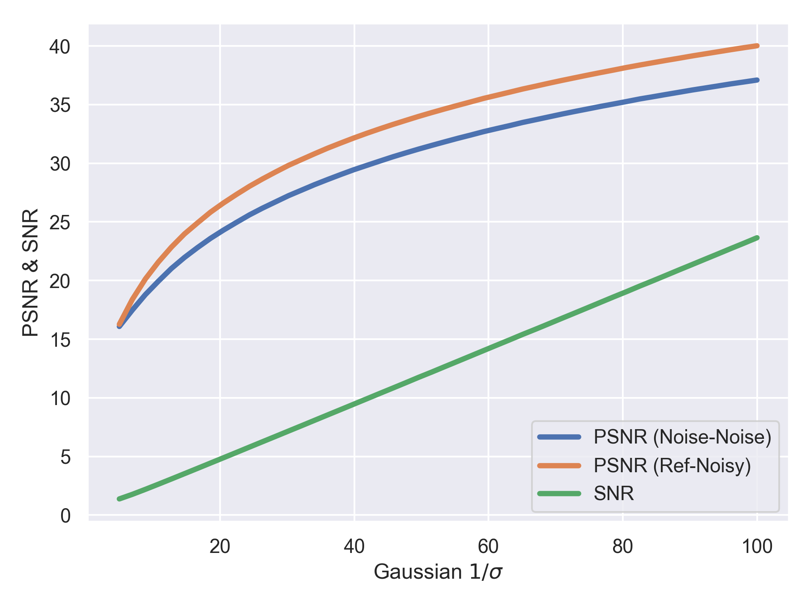


**Figure S4:** Simulation of PSNR calculations between two noisy images or one reference image for different noise levels, together with the standard dual acquisition SNR measurement.

## nnUNet Fiducial segmentation

### Training

A neural network based on the UNet design was trained to perform semantic segmentation of the 15 fiducials, used to quantify geometric distortion. The network design and training was performed using the nnUNet framework [6] with a residual encoder UNet design [7]. The training data was composed of 51 scans with three scans from each of the 17 sites, one in each orientation (axial, sagittal, and coronal). For each scan, the fiducials were manually segmented in their native space using 3Dslicer with a 10 mm 3D spherical mask [8]. Training was performed for each slice orientation separately, resulting in three dedicated networks. The network was trained using the nnUNetPlannerResEncM plan and the 3D full resolution configuration, with five folds. Since the nnUNet framework automatically finds the optimal training parameters, these will not be reported here. Training was performed over 1000 epochs on each fold separately. Each epoch took approximately 100 s, resulting in a total training time of 140 h. Training and inference were performed on the Lund University LUNARC cluster on a node equipped with an Nvidia A100 GPU and two Intel Xeon Gold 6226R 2.67 GHz 16-Core CPUs.

### Repeated scans validation

To validate the segmentation method of the geometric distortion fiducials, we compared the estimated fiducial positions between the two repeated scans in each exam in the dataset. We calculated the error ($\Delta$) between the estimated position after rigid body registration to the design space. **Figure S5** **A-C** show histograms of the error for each spatial direction, with each histogram clearly centred around 0 mm. The standard deviation was largest in the slice direction, as expected from the thick slices. The kurtosis was smallest in the frequency encoding direction. **Figure S5D** shows the kernel density estimates from the three histograms overlayed as a comparison of the distribution shapes. **Figure S6** shows the error in 2D (in the high-resolution plane) and 3D, showing larger mean error and standard deviation in 3D, as expected from the thick slices.


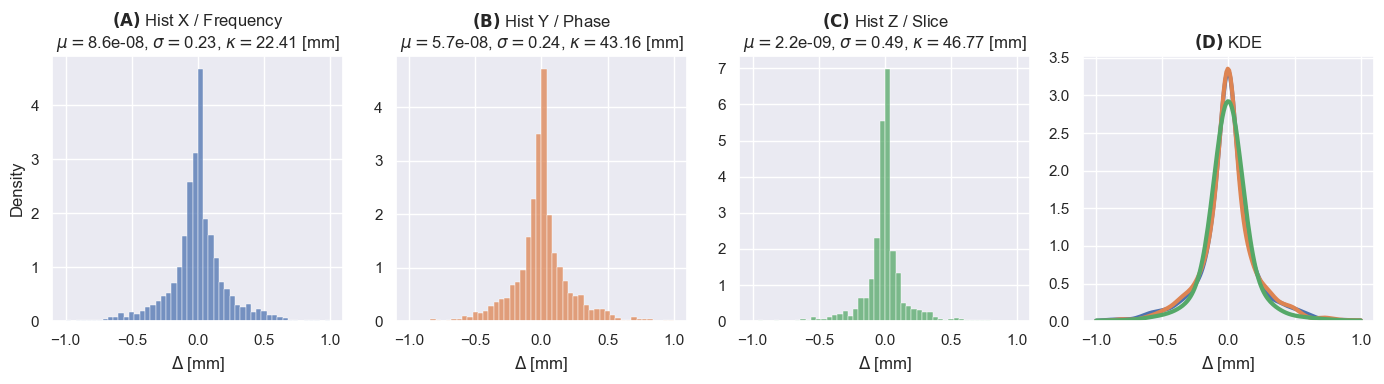


**Figure S5:** Difference in segmentation position between repeated axial scans for all subject for the different fiducials. Subplots **A**, **B**, and **C** show histograms of the segmentation error in the three spatial dimensions, calculated with 50 bins in the range [-1,1]. Subplot **D** shows the corresponding kernel density estimate (KDE) of the three histograms.


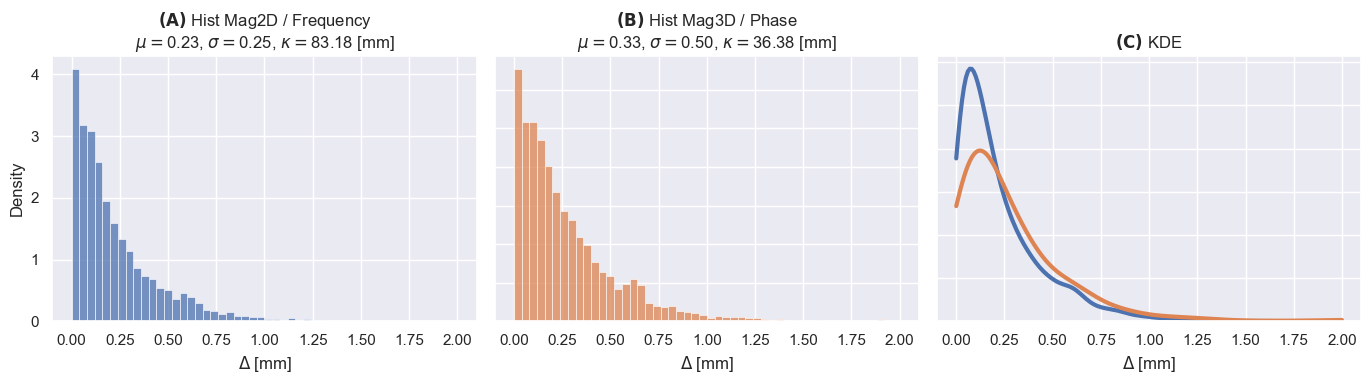


**Figure S6:** Difference in segmentation between the two repeated axial scans calculated in 2D (in-plane) **(A)** and 3D **(B)**. Subplot **C** shows the corresponding kernel density estimate (KDE).

# Relaxometry results

**Figure S7** shows a comparison of the T_1_ and T_2_ values obtained from this study (P0004) to the relaxometry values obtained at 3 T from NIST. The T_1_ values of the NiCl_2_ is almost identical between 64 mT and 3 T, indicating by following the line of unity in dashed black. **Tables S3**, **S4**, and **S5** lists the measured T_1_ and T_2_ values for the three arrays of tissue mimics.


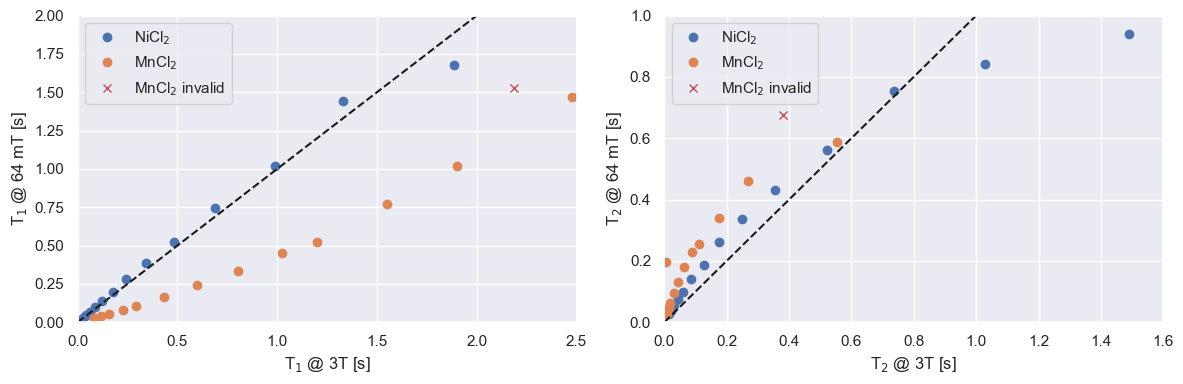


**Figure S7**: Comparison of T_1_ and T_2_ values at 64 mT (from P0004 in this study) and reference values at 3 T from NIST at 20°C. MnCl_2_ mimic 2 was excluded from the analysis due to known drift as reported by the manufacturer, here shown as red cross. Dashed black line indicates a 1:1 relation between the two field strengths.

**Table S4:** NiCl_2_ mimics relaxometry results. Mimic 14 was not included in the calculation of the relaxivity and therefore marked in red.

| Mimic | Content | Conc. [mM] | T_1_ [s] | T_2_ [s] |
| --- | --- | --- | --- | --- |
| 1 | NiCl_2_ | 0.29 | 1.679 | 0.940 |
| 2 | NiCl_2_ | 0.6 | 1.440 | 0.842 |
| 3 | NiCl_2_ | 1.04 | 1.019 | 0.753 |
| 4 | NiCl_2_ | 1.64 | 0.742 | 0.563 |
| 5 | NiCl_2_ | 2.52 | 0.524 | 0.431 |
| 6 | NiCl_2_ | 3.68 | 0.383 | 0.338 |
| 7 | NiCl_2_ | 5.43 | 0.279 | 0.262 |
| 8 | NiCl_2_ | 7.74 | 0.200 | 0.187 |
| 9 | NiCl_2_ | 11.3 | 0.141 | 0.141 |
| 10 | NiCl_2_ | 16.5 | 0.097 | 0.100 |
| 11 | NiCl_2_ | 23.3 | 0.069 | 0.076 |
| 12 | NiCl_2_ | 32.7 | 0.049 | 0.055 |
| 13 | NiCl_2_ | 46 | 0.034 | 0.037 |
| 14 | NiCl_2_ | 65.3 | 0.024 | 0.028 |

**Table S5:** MnCl_2_ mimics relaxometry results. Mimics 1, 2, 13, and 14 were not included in the relaxivity calculation and therefore marked in red. Mimic 2 was also excluded due to drift reported by the manufacturer.

| Mimic | Content | Conc. [mM] | T_1_ [s] | T_2_ [s] |
| --- | --- | --- | --- | --- |
| 1 | MnCl_2_ | 0.0113 | 1.468 | 0.589 |
| 2 | MnCl_2_ | 0.0282 | 1.016 | 0.459 |
| 3 | MnCl_2_ | 0.0434 | 0.768 | 0.339 |
| 4 | MnCl_2_ | 0.0673 | 0.523 | 0.256 |
| 5 | MnCl_2_ | 0.0934 | 0.449 | 0.228 |
| 6 | MnCl_2_ | 0.1353 | 0.336 | 0.180 |
| 7 | MnCl_2_ | 0.193 | 0.244 | 0.131 |
| 8 | MnCl_2_ | 0.2768 | 0.165 | 0.095 |
| 9 | MnCl_2_ | 0.4276 | 0.108 | 0.064 |
| 10 | MnCl_2_ | 0.5555 | 0.082 | 0.050 |
| 11 | MnCl_2_ | 0.7902 | 0.056 | 0.035 |
| 12 | MnCl_2_ | 1.1274 | 0.042 | 0.027 |
| 13 | MnCl_2_ | 1.5996 | 0.029 | 0.197 |

**Table S6**: Diffusion mimics relaxometry results with NIST-certified ADC values at 21 °C, 3 T.

| Mimic | Content | Conc. [mM] | T_1_ [s] | T_2_ [s] | ADC ($\boldsymbol{\mu}\mathbf{m}^{\boldsymbol{2}}\boldsymbol{/}\mathbf{s}$) |
| --- | --- | --- | --- | --- | --- |
| 1 | PVP | 0 | 2.37 | 1.53 | 2067 |
| 2 | PVP | 50 | 0.29 | 0.27 | 315 |
| 3 | PVP | 0 | 2.35 | 1.53 | 2067 |
| 4 | PVP | 0 | 2.38 | 1.30 | 2067 |
| 5 | PVP | 10 | 1.71 | 1.13 | 1591 |
| 6 | PVP | 20 | 1.28 | 1.10 | 1211 |
| 7 | PVP | 30 | 0.93 | 0.94 | 876 |
| 8 | PVP | 40 | 0.54 | 0.47 | 527 |
| 9 | PVP | 40 | 0.54 | 0.53 | 527 |
| 10 | PVP | 50 | 0.30 | 0.29 | 315 |
| 11 | PVP | 0 | 2.51 | 1.86 | 2067 |
| 12 | PVP | 10 | 1.82 | 1.56 | 1591 |
| 13 | PVP | 20 | 1.27 | 1.13 | 1211 |
| 14 | PVP | 30 | 0.92 | 0.92 | 876 |

# References

1. Bertini I, Luchinat C, Parigi G (2005) 1H NMRD PROFILES OF PARAMAGNETIC COMPLEXES AND METALLOPROTEINS. Advances in Inorganic Chemistry. Elsevier, pp 105–172

2. Martin MN, Jordanova KV, Kos AB, Russek SE, Keenan KE, Stupic KF (2023) Relaxation measurements of an MRI system phantom at low magnetic field strengths. Magn Reson Mater Phy. doi: 10.1007/s10334-023-01086-y

3. Stupic KF, Ainslie M, Boss MA, Charles C, Dienstfrey AM, Evelhoch JL, Finn P, Gimbutas Z, Gunter JL, Hill DLG, Jack CR, Jackson EF, Karaulanov T, Keenan KE, Liu G, Martin MN, Prasad PV, Rentz NS, Yuan C, Russek SE (2021) A standard system phantom for magnetic resonance imaging. Magnetic Resonance in Med 86:1194–1211.

4. Lowekamp BC, Chen DT, Ibáñez L, Blezek D (2013) The Design of SimpleITK. Front Neuroinform. doi: 10.3389/fninf.2013.00045

5. Tustison NJ, Cook PA, Holbrook AJ, Johnson HJ, Muschelli J, Devenyi GA, Duda JT, Das SR, Cullen NC, Gillen DL, Yassa MA, Stone JR, Gee JC, Avants BB (2021) The ANTsX ecosystem for quantitative biological and medical imaging. Sci Rep 11:9068.

6. Isensee F, Jaeger PF, Kohl SAA, Petersen J, Maier-Hein KH (2021) nnU-Net: a self-configuring method for deep learning-based biomedical image segmentation. Nat Methods 18:203–211.

7. Isensee F, Wald T, Ulrich C, Baumgartner M, Roy S, Maier-Hein K, Jaeger PF (2024) nnU-Net Revisited: A Call for Rigorous Validation in 3D Medical Image Segmentation.

8. Fedorov A, Beichel R, Kalpathy-Cramer J, Finet J, Fillion-Robin J-C, Pujol S, Bauer C, Jennings D, Fennessy F, Sonka M, Buatti J, Aylward S, Miller JV, Pieper S, Kikinis R (2012) 3D Slicer as an image computing platform for the Quantitative Imaging Network. Magnetic Resonance Imaging 30:1323–1341.
